# Supplementary material for: Cancer lineage-specific regulation of YAP responsive elements revealed through large-scale functional epigenomic screens
Source: Nat Commun. 2023 Jul 3;14:3907. doi: 10.1038/s41467-023-39527-w (PMC10317959; doi:10.1038/s41467-023-39527-w)
Supplement: Supplementary file 1 — Supplementary Information [file 41467_2023_39527_MOESM1_ESM.pdf]

Supplementary Information

Cancer lineage-specific regulation of YAP responsive elements revealed through large-scale functional epigenomic screens

Supplementary Figure Legends:

Figure S1. YAP engages enhancer elements in both UM and MPM

A. RT-qPCR quantification of YAP1 and CYR61 expression in mesothelioma (NCI-H2052 and MSTO-211H) and uveal melanoma (92.1 and MEL202) cell lines expressing dox-inducible shRNAs against a neutral control (sh.Neutral) or against YAP1 (shYAP1.1741 and shYAP1.2371). **B.** Heatmap representing genome-wide correlation of TMM normalized read counts of H3K27ac, H3K4me1, H3K4me3 and RPB1 ChIP-seq in mesothelioma and uveal melanoma cell lines. **C.** Heatmaps showing ChIP-seq signal of YAP1, TAZ, TEAD4, RPB1, H3K27ac, H3K4me1, and H3K4me3 centered around YAP1 ChIP-seq peak summits in NCI-H2052, 92.1 and OMM1 cell lines. The peak summits have been clustered by their H3K4me3, H3K4me1, and RPB1 signal. The summits have been arranged in decreasing order of YAP1 signal within each cluster.

Figure S2. Design and features of YMCi-160K library

A. Flowchart detailing the design steps of the YMCi-160K CRISPRi library. **B.** Pie chart and table summarizing the percentage and number of sgRNAs within the YMCi-160K library that target YAP1 peaks present in all cell lines (yellow color), subsets of cell lines (green, purple, black, or dark grey) or each individual cell line (light grey). Within these classes, the number of YAP1 peaks targeted by these sgRNAs is also shown. **C.** Bar graph showing the total number of YAP1 peaks targeted in each cell line. **D.** Bar graph showing the distribution of number of sgRNAs per YAP peak, in pool 1 (red color) or pool 2 (blue color). In both pools, the majority of peaks are targeted by 10 sgRNAs. **E.** Histogram representing the distance between sgRNAs within a YAP peak summit, in pool 1 (red color) or pool 2 (blue color). In both pools, each sgRNA distances approximately 61 bp from its neighbouring sgRNA. **F.** Graph showing the even distribution of sgRNAs around YAP peak summits. sgRNAs are contained within a +/- 300bp window around the peak summit.

Figure S3. Quality control for YMCi-160K screens

A. Scatter plots showing the correlation between pool 1 and pool2 log2-fold changes for the various control-guides subgroups (NT – non-targeting; PL – pan-lethal; HS – hippo signaling genes; YT – YAP/TEAD targets) across timepoints, in both mesothelioma (NCI-H2052) and uveal melanoma (92.1) YMCi-160K screens. **B.** Scatter plots showing correlation of TMM normalized counts of experimental-guides between

the two replicates performed for each screen in mesothelioma (NCI-H2052) and uveal melanoma (92.1) cell lines.

Figure S4. Comparative analyses between UM and MPM YMCi-160K screen results

A. Bar graph showing the abundance (Log2-fold change) of sgRNAs targeting the promoter region of YAP1, TAZ, TEAD1, TEAD2, TEAD3 and TEAD4, at the three timepoints (day 8, day 15 and day 22) of the mesothelioma (NCI-H2052) and uveal melanoma (92.1) screens. **B.** Scatter plot of Log2-fold change at day 22 of sgRNAs targeting YAP/TEAD target genes (left plot) or Hippo pathway components (right plot) in mesothelioma (NCI-H2052) versus uveal melanoma (92.1) screens. Canonical YAP/TEAD target genes (AMOTL2, CTGF, CYR61 and ANKRD1) are highlighted in red. **C.** Scatter plot comparing mesothelioma (NCI-H2052) versus uveal melanoma (92.1) sgRNA abundance (Log2-fold change) at day 22. sgRNAs selected for inclusion in YMCi-13K are colored: yellow for sgRNAs scoring in both cell lines, green for mesothelioma-specific and purple for uveal melanoma-specific. sgRNAs not significantly scoring in YMCi-160K (hence not selected for secondary-screen validation) are depicted in light grey. **D.** Volcano plot showing the comparison of sgRNA depletion between H2052 (MPM) and 92.1 (UM) cells at day 8 (left panel) and day 15 (right panel). Significantly depleted sgRNAs (FDR <0.05) are colored (NCI-H2052, green dots; 92.1, purple dots). Grey dots depict non-differentially represented sgRNAs. X-axis represents Log2(Fold change) between the two cell lines. Y-axis depicts the log10-transformed FDR value. **E.** Quantification of YAP-peaks targeted by the non-scoring or scoring sgRNAs shown in panel S4C, for each category: 400 peaks score specifically in mesothelioma, 800 peaks score specifically in uveal melanoma and 1800 peaks score in both lineages.

Figure S5. Quality control for YMCi-13K screens

A. Distribution of log2-fold change values for sgRNAs within the various control and experimental categories, at day 8, day 15 and day 22 timepoints compared to day 0, in both mesothelioma (NCI-H2052 and MSTO-211H) and uveal melanoma (92.1 and MEL202) screens. Significance of depletion is represented as -Log10 FDR in grey colored scale. **B.** Comparison of sgRNA abundance (log2-fold change) between primary (YMCi-160K) and secondary (YMCi-13K) screens of each cell line, at days 8, 15 or 22 compared to day 0. Highlighted in green (mesothelioma – NCI-H2052) or purple (uveal melanoma – 92.1) color are sgRNAs with significant depletion (defined as Log2 FC < -1 and FDR < 0.01) in both primary and secondary screens for each cell line. **C.** Heatmap depicting correlation of TMM normalized counts from plasmid pool (day 0) and secondary screens in mesothelioma (NCI-H2052 and MSTO-211H) and uveal melanoma (92.1 and MEL202) cell lines at three different timepoints (day 8, day 15 and day 22). **D.** Bar graphs showing the abundance (Log2-fold change) of sgRNAs targeting the promoter region of YAP1, TAZ, TEAD1, TEAD2, TEAD3 and TEAD4, at the three timepoints (day 8, day 15 and day 22) in mesothelioma (NCI-H2052 and MSTO-211H) and uveal melanoma (92.1 and MEL202) secondary screens.

Figure S6. Hits validation of YMCi-13K screens

A. Visual example of FACS gating strategy for competitive proliferation assays. **B.** Competitive proliferation assays in mesothelioma (NCI-H2052 and MSTO-211H) or uveal melanoma (92.1 and MEL202) CRISPRi cells transduced with sgRNA controls (non-targeting or targeting the essential gene RPL14) or with multiple sgRNAs targeting the TSS of YAP1. **C.** RT-qPCR quantification of the mRNA of YAP1 and its canonical target genes AMOTL2, CTGF and CYR61 in mesothelioma (NCI-H2052 and MSTO-211H) and uveal melanoma (92.1 and MEL202) CRISPRi cell lines transduced with non-targeting sgRNAs (sgNTC) or three sgRNAs targeting the TSS of YAP1. **D.** Competitive proliferation assays in CRISPRi mesothelioma (NCI-H2052 and MSTO-211H) or uveal melanoma (92.1 and MEL202) cells transduced with sgRNA targeting mesothelioma-specific hits – TSS of NDC1, TSS of AAAS or a putative NR2F2 enhancer. **E.** Competitive proliferation assays in CRISPRi mesothelioma (NCI-H2052 and MSTO-211H) or uveal melanoma (92.1 and MEL202) cells transduced with sgRNAs targeting uveal melanoma-specific hits – TSS of NR4A3 or TSS of KEAP1. For all competitive proliferation assays, the percentage of sgRNA expressing cells (RFP+ cells) was monitored by flow cytometry for 31 days and values were normalized to day 3 after-transduction. **F**. RT-qPCR quantification of the mRNA of lineage-specific hits analyzed in C and D in mesothelioma (NCI-H2052 and MSTO-211H) and uveal melanoma (92.1 and MEL202) CRISPRi cell lines transduced with two control sgRNAs (NTC, non-targeting) or the indicated sgRNAs targeting lineage-specific hits.

Figure S7. Validation of hits at the MYC locus

A. Genome browser snapshot of the MYC locus (chr8:127,680,000-129,735,000) with tracks representing: ChIP-seq YAP peak summits (red bars), scoring regions in YMCi screens, representation at day 22 (log2-fold change) of single sgRNAs from the primary YMCi-160K screens in mesothelioma (NCI-H2052; green color) and uveal melanoma (92.1; purple color), and Cut&Tag tracks for YAP1 and H3K27ac. **B.** Competitive proliferation assays in CRISPRi mesothelioma (NCI-H2052 and MSTO-211H) or uveal melanoma (92.1 and MEL202) cells transduced with sgRNA targeting the TSS of MYC or the lineage-specific scoring regions +440Kb (mesothelioma) or +1800Kb (uveal melanoma) from MYC TSS. **C.** RT-qPCR quantification of MYC expression levels in mesothelioma (NCI-H2052 and MSTO-211H) and uveal melanoma (92.1 and MEL202) CRISPRi cell lines transduced with non-targeting control sgRNAs (NTC), sgRNAs against the MYC TSS, or sites +440Kb or 1800Kb from the MYC TSS.

Figure S8. Evaluation of functional YREs in FOSL1 and FOSB loci

A. Genome browser snapshot of the FOSL1 and FOSB loci showing YMCi-13K screen results (scoring regions and Log2-fold change at day 22 using bigwig tracks with 250bp span), HiC data, HiChIP-H3K27ac loops, Cut&Tag tracks for YAP1, FOSL1, JUN and CTCF, and ChIP-seq tracks for H3K27ac, H3K4me1, H3K4me3 and the RNA polymerase II subunit RPB1. Scoring regions validated in single assays are color-shaded in green.

Figure S9. YREs engagement of MAPK TFs in MPM

A. Competitive proliferation assays in CRISPRi mesothelioma (NCI-H2052 and MSTO-211H) or uveal melanoma (92.1 and MEL202) cells transduced with sgRNAs targeting two MPM-specific scoring regions: -361Kb upstream of JUN TSS (green lines) or +10Kb downstream of FOSB TSS (brown lines). **B.** Competitive proliferation assays in CRISPRi mesothelioma (NCI-H2052 and MSTO-211H) or uveal melanoma (92.1 and MEL202) cells transduced with sgRNAs targeting the TSS of FOSL1 or the MPM-specific scoring regions -3Kb or -15Kb from FOSL1 TSS. **C.** RT-qPCR quantification of JUN expression levels in mesothelioma (NCI-H2052 and MSTO-211H) or uveal melanoma (92.1 and MEL202) CRISPRi cells transduced with non-targeting control sgRNAs (sgNTC), or sgRNAs against the enhancer region -361Kb from JUN TSS. **D.** RT-qPCR quantification of FOSB expression levels in mesothelioma (NCI-H2052 and MSTO-211H) or uveal melanoma (92.1 and MEL202) CRISPRi cells transduced with control sgRNAs (sgNTC), or sgRNAs against the enhancer region +10Kb from FOSB TSS. **E.** RT-qPCR quantification of FOSL1 expression levels in mesothelioma (NCI-H2052 and MSTO-211H) CRISPRi cell lines transduced with control sgRNAs (sgNTC), or sgRNAs against the TSS of FOSL1, or the enhancer regions -3Kb and -15Kb from the FOSL1 TSS. FOSL1 expression was not detected in the uveal melanoma cell lines used in this study (data not shown). Gene expression levels were normalized to GAPDH in all RT-qPCR experiments. **F.** Heatmap showing Spearman-rank correlation of CTCF, YAP, PAX3, SOX10, MITF, TFAP2A, JUN and FOSL1 Cut&Tag read counts around the union of YAP summits (+/- 250bp radius), in mesothelioma (NCI-H2052 and MSTO-211H) and uveal melanoma (92.1 and MEL202) cell lines. **G.** Expression levels from TCGA dataset of transcription factors involved in MAPK signaling in patient samples of mesothelioma (green color), uveal melanoma (purple color) or other tumor origin (grey color).

Figure S10. Evaluation of functional YREs in PAX3 and MITF loci

A. Competitive proliferation assay in CRISPRi mesothelioma (NCI-H2052 and MSTO-211H) or uveal melanoma (92.1 and MEL202) cells transduced with sgRNAs targeting the TSS of SOX10 or the UM-specific scoring region -54Kb from SOX10 TSS. **B.** RT-qPCR quantification of SOX10 and POLR2F mRNA levels in uveal melanoma (92.1 and MEL202) and mesothelioma (NCI-H2052 and MSTO-211H) cells transduced with non-targeting control sgRNAs (sgNTC), or sgRNAs against the TSS of SOX10, or the enhancer region -54Kb from SOX10 TSS. **C.** Genome browser snapshot of the PAX3 and MITF loci showing YMCi-13K screen results (scoring regions and Log2-fold change at day 22 using bigwig tracks with 250bp span), HiC data, HiChIP-H3K27ac loops, Cut&Tag tracks for YAP1, PAX3, SOX10, MITF, TFAP2A and CTCF, and ChIP-seq tracks for H3K27ac, H3K4me1, H3K4me3 and the RNA polymerase II subunit RPB1. Scoring regions validated in single assays are color-shaded in purple. **D.** Competitive proliferation assay in CRISPRi mesothelioma (NCI-H2052 and MSTO-211H) or uveal melanoma (92.1 and MEL202) cells transduced with sgRNAs against the TSS of PAX3 or the enhancer region +138Kb from PAX3 TSS. **E.** Competitive proliferation assay in CRISPRi mesothelioma (NCI-H2052 and MSTO-211H) or uveal melanoma (92.1 and MEL202) cells transduced with sgRNAs against the TSS of MITF or the

enhancer regions -97Kb or -115Kb from MITF TSS. **F.** RT-qPCR quantification of PAX3 expression levels in uveal melanoma (92.1 and MEL202) CRISPRi cells transduced with non-targeting control sgRNAs (sgNTC), or sgRNAs against the enhancer region +138Kb from PAX3 TSS. **G.** RT-qPCR quantification of MITF expression levels in uveal melanoma (92.1 and MEL202) CRISPRi cells transduced with control sgRNAs (sgNTC), or sgRNAs against the TSS of MITF or the enhancer regions -97Kb or -115Kb from MITF TSS. Expression of PAX3 and MITF was not detected in the mesothelioma cell lines used (data not shown). Gene expression levels were normalized to GAPDH in all RT-qPCR experiments.

Figure S11. Evaluation of Melanocytic TFs expression and dependency in large-scale datasets
A. Expression levels from TCGA dataset of transcription factors associated with the melanocytic lineage, in patient samples of mesothelioma (green color), uveal melanoma (purple color) or other cancer tissue origin (grey color). **B.** Box plots displaying the sensitivity to CRISPR KO of PAX3, SOX10, MITF and TFAP2A in cell lines of mesothelioma origin (green color), uveal melanoma origin (purple color) or from other malignancies (grey color). Analysis was performed using data from DEPMAP.

Figure S1

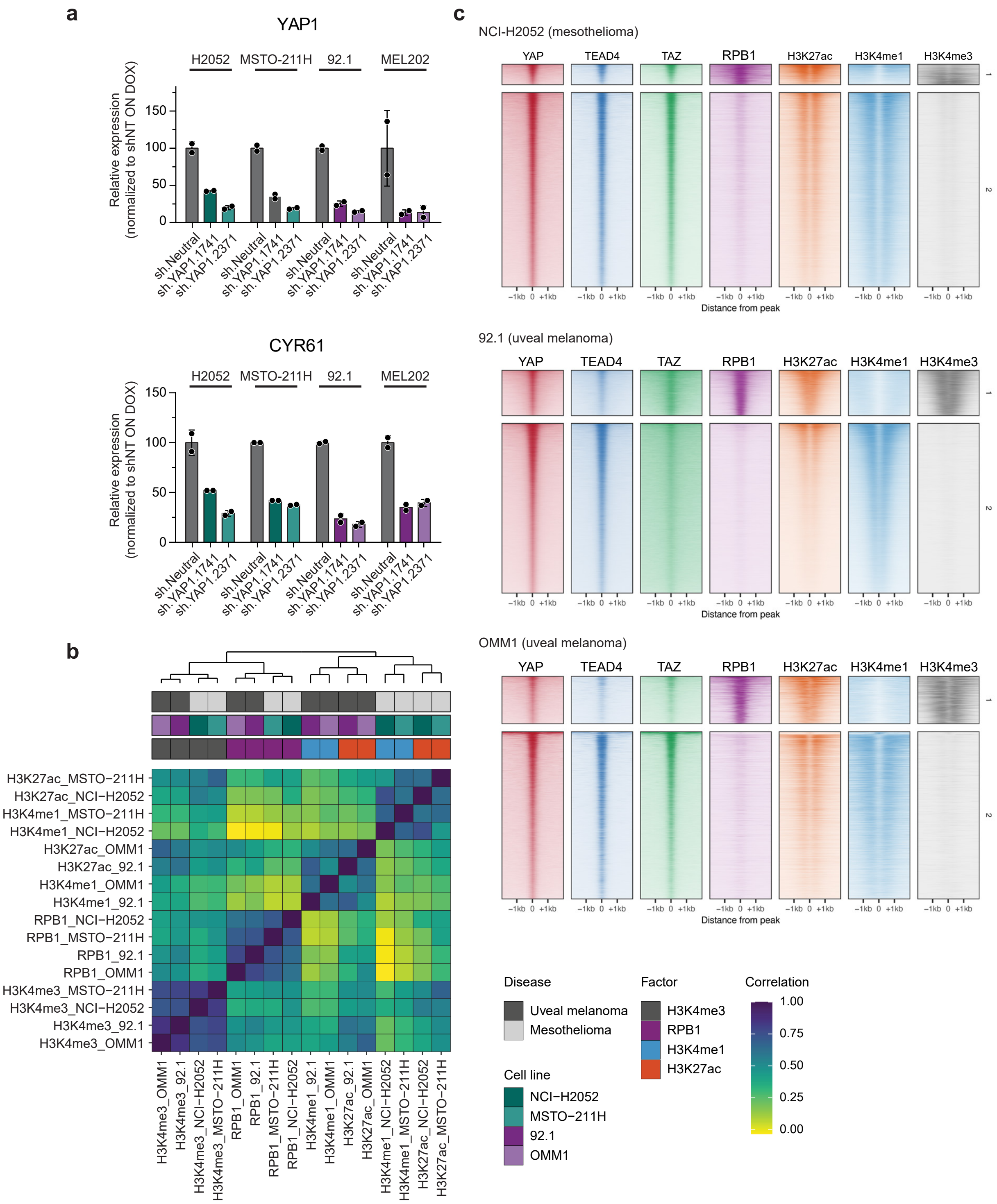
a
YAP1
H2052
MSTO-211H
92.1
MEL202
Relative expression
(normalized to shNT ON DOX)
150
100
50
0
sh.Neutral
sh.YAP1.1741
sh.YAP1.2371
CYR61
b
H3K27ac_MSTO−211H
H3K27ac_NCI−H2052
H3K4me1_MSTO−211H
H3K4me1_NCI−H2052
H3K27ac_OMM1
H3K27ac_92.1
H3K4me1_OMM1
H3K4me1_92.1
RPB1_NCI−H2052
RPB1_MSTO−211H
RPB1_92.1
RPB1_OMM1
H3K4me3_MSTO−211H
H3K4me3_NCI−H2052
H3K4me3_92.1
H3K4me3_OMM1
c
NCI-H2052 (mesothelioma)
92.1 (uveal melanoma)
OMM1 (uveal melanoma)
YAP
TEAD4
TAZ
RPB1
H3K27ac
H3K4me1
H3K4me3
−1kb 0 +1kb
Distance from peak
Disease
Uveal melanoma
Mesothelioma
Cell line
NCI−H2052
MSTO−211H
92.1
OMM1
Factor
H3K4me3
RPB1
H3K4me1
H3K27ac
Correlation
1.00
0.75
0.50
0.25
0.00

Figure S2

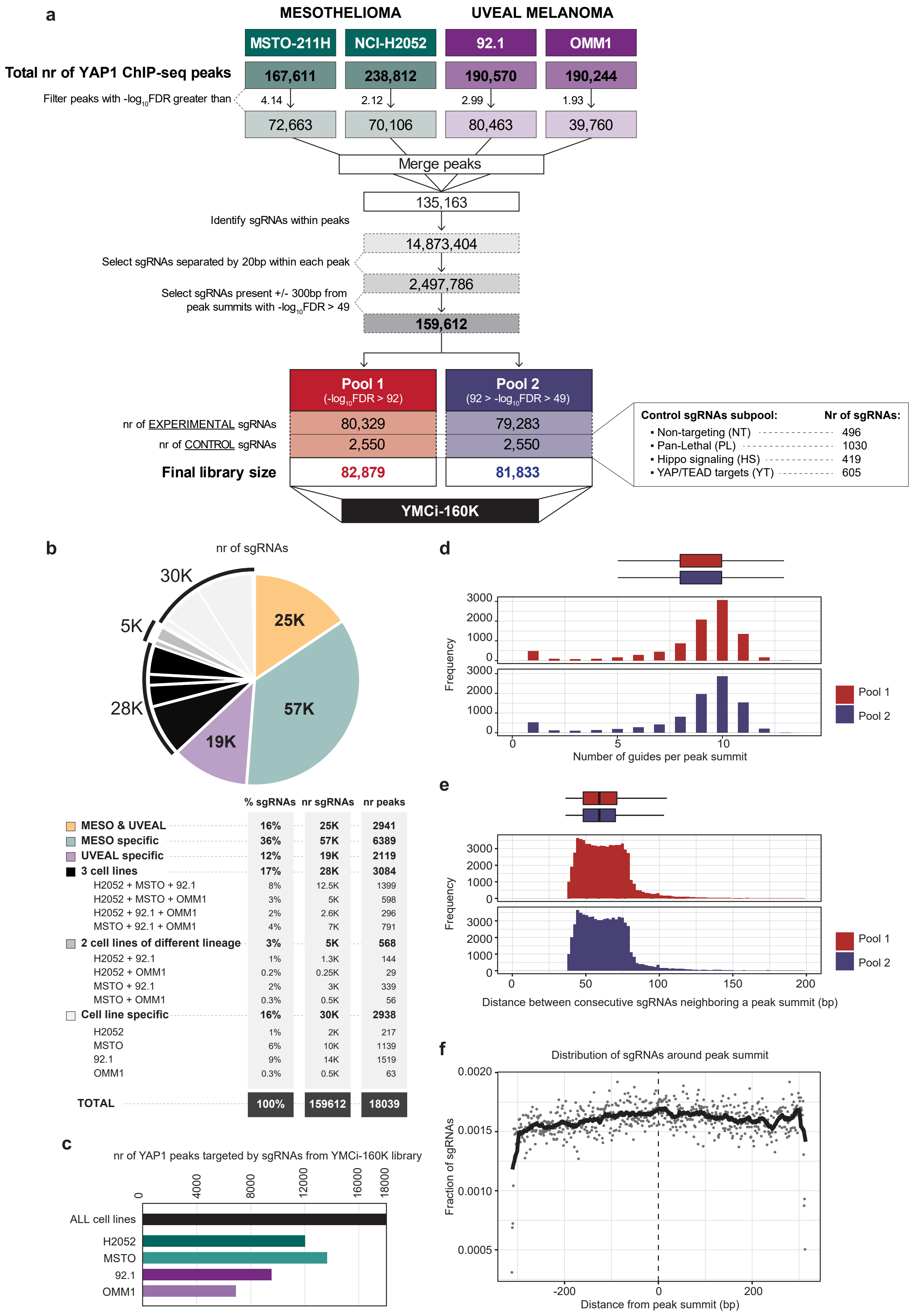
a
MESOTHELIOMA
UVEAL MELANOMA
MSTO-211H
NCI-H2052
92.1
OMM1
Total nr of YAP1 ChIP-seq peaks
167,611
238,812
190,570
190,244
Filter peaks with -log10FDR greater than
4.14
2.12
2.99
1.93
72,663
70,106
80,463
39,760
Merge peaks
135,163
Identify sgRNAs within peaks
14,873,404
Select sgRNAs separated by 20bp within each peak
2,497,786
Select sgRNAs present +/- 300bp from peak summits with -log10FDR > 49
159,612
Pool 1
(-log10FDR > 92)
Pool 2
(92 > -log10FDR > 49)
nr of EXPERIMENTAL sgRNAs
80,329
79,283
nr of CONTROL sgRNAs
2,550
2,550
Final library size
82,879
81,833
YMCi-160K
Control sgRNAs subpool:
Nr of sgRNAs:
• Non-targeting (NT) 496
• Pan-Lethal (PL) 1030
• Hippo signaling (HS) 419
• YAP/TEAD targets (YT) 605
b
nr of sgRNAs
30K
5K
28K
25K
57K
19K
% sgRNAs
nr sgRNAs
nr peaks
MESO & UVEAL 16% 25K 2941
MESO specific 36% 57K 6389
UVEAL specific 12% 19K 2119
3 cell lines 17% 28K 3084
H2052 + MSTO + 92.1 8% 12.5K 1399
H2052 + MSTO + OMM1 3% 5K 598
H2052 + 92.1 + OMM1 2% 2.6K 296
MSTO + 92.1 + OMM1 4% 7K 791
2 cell lines of different lineage 3% 5K 568
H2052 + 92.1 1% 1.3K 144
H2052 + OMM1 0.2% 0.25K 29
MSTO + 92.1 2% 3K 339
MSTO + OMM1 0.3% 0.5K 56
Cell line specific 16% 30K 2938
H2052 1% 2K 217
MSTO 6% 10K 1139
92.1 9% 14K 1519
OMM1 0.3% 0.5K 63
TOTAL 100% 159612 18039
c
nr of YAP1 peaks targeted by sgRNAs from YMCi-160K library
0
4000
8000
12000
16000
18000
ALL cell lines
H2052
MSTO
92.1
OMM1
d
Frequency
3000
2000
1000
0
0
5
10
Number of guides per peak summit
Pool 1
Pool 2
e
Frequency
3000
2000
1000
0
0
50
100
150
200
Distance between consecutive sgRNAs neighboring a peak summit (bp)
Pool 1
Pool 2
f
Distribution of sgRNAs around peak summit
Fraction of sgRNAs
0.0020
0.0015
0.0010
0.0005
-200
0
200
Distance from peak summit (bp)

Figure S3

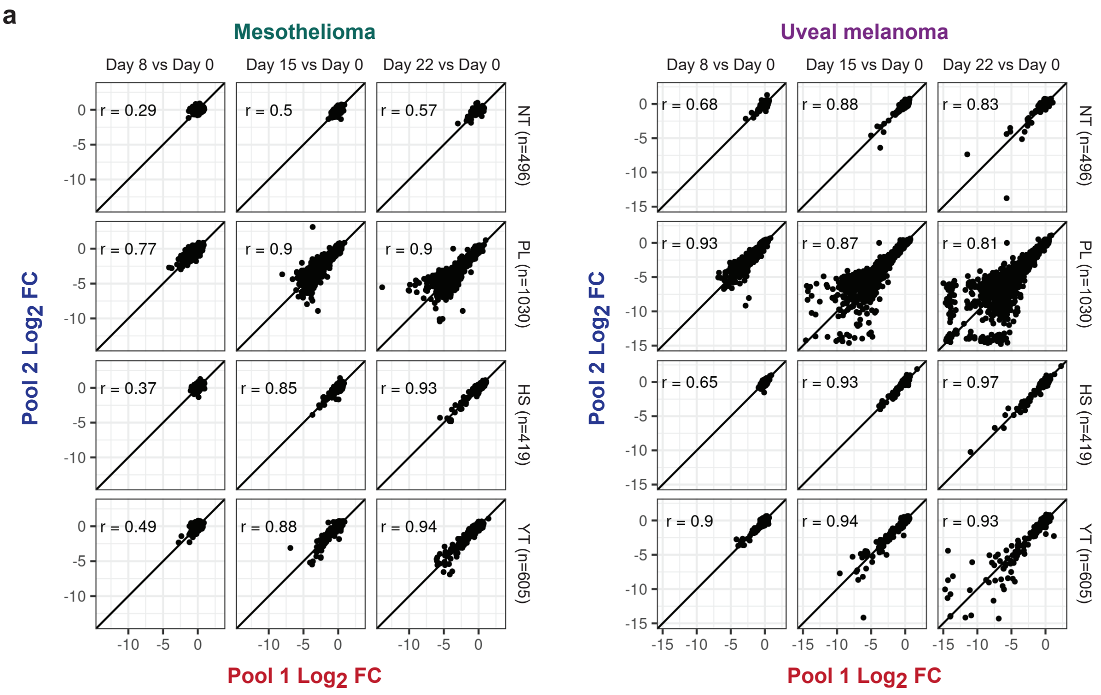

a
Mesothelioma
Uveal melanoma
Day 8 vs Day 0
Day 15 vs Day 0
Day 22 vs Day 0
NT (n=496)
PL (n=1030)
HS (n=419)
YT (n=605)
r = 0.29
r = 0.5
r = 0.57
r = 0.77
r = 0.9
r = 0.9
r = 0.37
r = 0.85
r = 0.93
r = 0.49
r = 0.88
r = 0.94
r = 0.68
r = 0.88
r = 0.83
r = 0.93
r = 0.87
r = 0.81
r = 0.65
r = 0.93
r = 0.97
r = 0.9
r = 0.94
r = 0.93
Pool 2 Log2 FC
Pool 1 Log2 FC

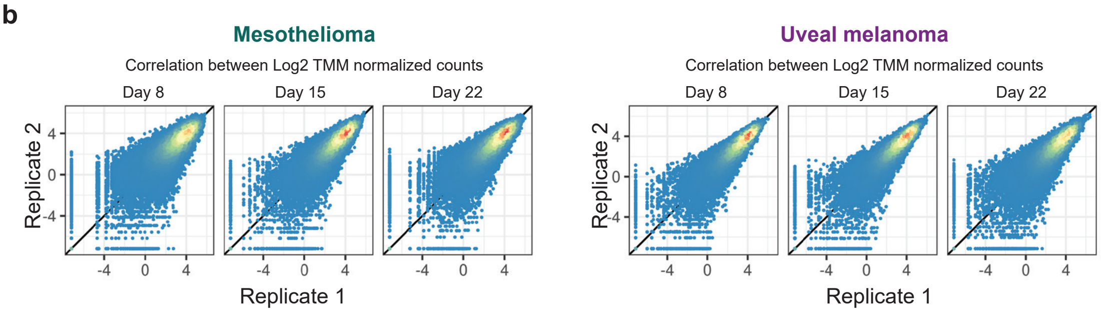

b
Mesothelioma
Uveal melanoma
Correlation between Log2 TMM normalized counts
Day 8
Day 15
Day 22
Replicate 2
Replicate 1

Figure S4

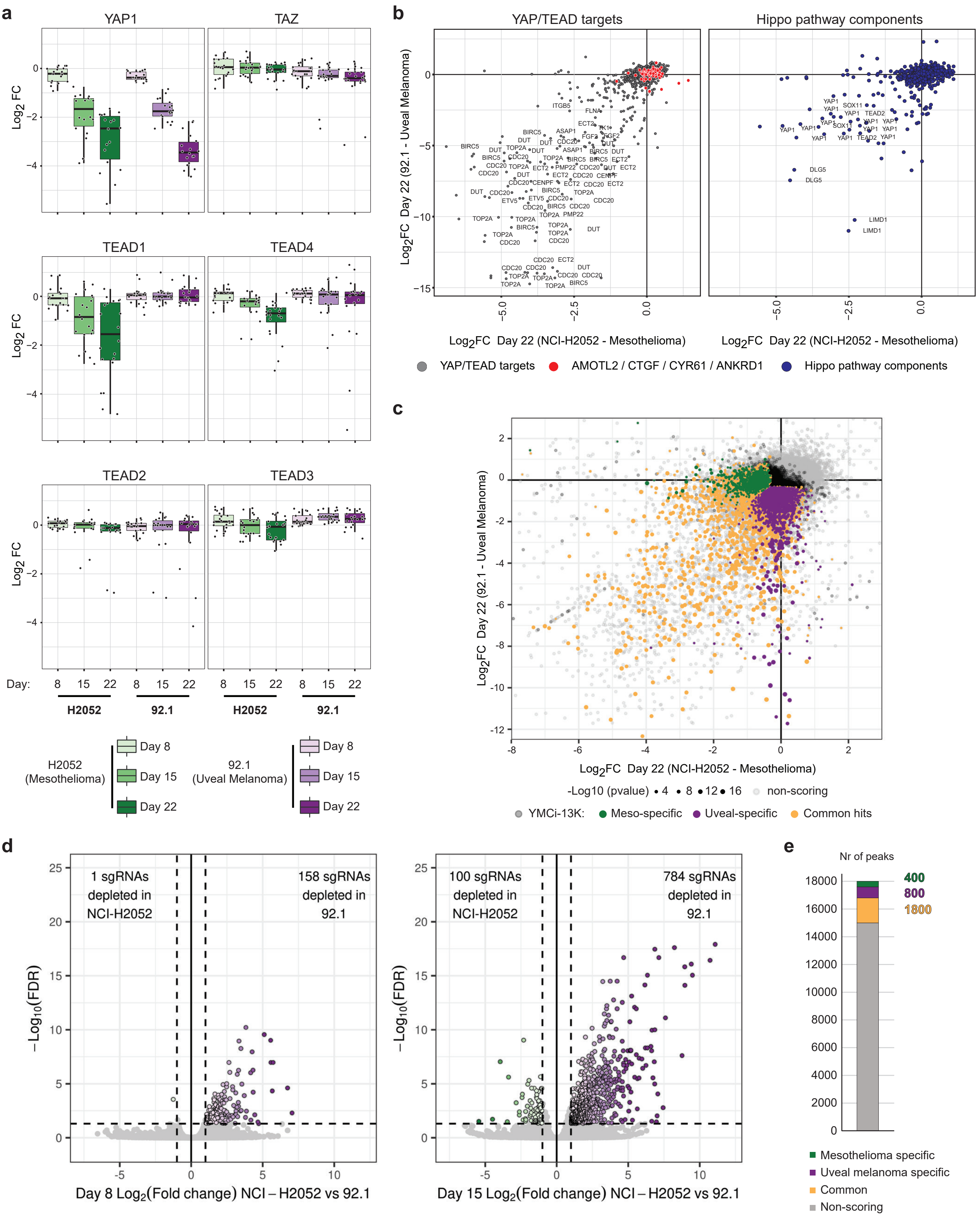
a
YAP1
TAZ
TEAD1
TEAD4
TEAD2
TEAD3
Log2 FC
Day: 8 15 22 8 15 22 8 15 22 8 15 22
H2052
92.1
H2052 (Mesothelioma)
Day 8
Day 15
Day 22
92.1 (Uveal Melanoma)
b
YAP/TEAD targets
Hippo pathway components
Log2FC Day 22 (92.1 - Uveal Melanoma)
Log2FC Day 22 (NCI-H2052 - Mesothelioma)
YAP/TEAD targets
AMOTL2 / CTGF / CYR61 / ANKRD1
Hippo pathway components
c
Log2FC Day 22 (92.1 - Uveal Melanoma)
Log2FC Day 22 (NCI-H2052 - Mesothelioma)
-Log10 (pvalue) • 4 • 8 • 12 • 16 non-scoring
YMCi-13K: Meso-specific Uveal-specific Common hits
d
1 sgRNAs depleted in NCI-H2052
158 sgRNAs depleted in 92.1
−Log10(FDR)
Day 8 Log2(Fold change) NCI – H2052 vs 92.1
100 sgRNAs depleted in NCI-H2052
784 sgRNAs depleted in 92.1
Day 15 Log2(Fold change) NCI – H2052 vs 92.1
e
Nr of peaks
400
800
1800
Mesothelioma specific
Uveal melanoma specific
Common
Non-scoring

Figure S5

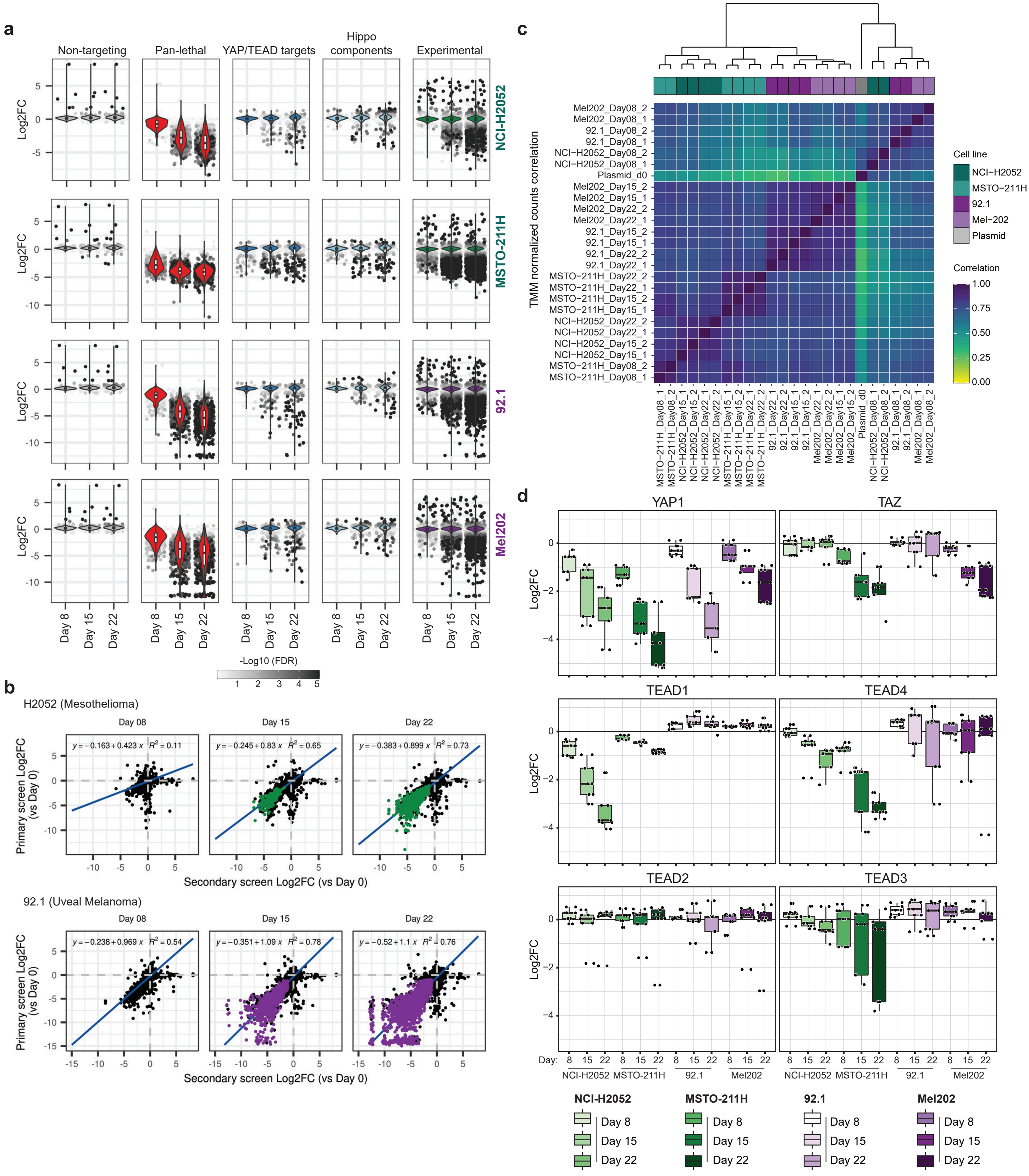

a
Non-targeting
Pan-lethal
YAP/TEAD targets
Hippo components
Experimental
Log2FC
NCI-H2052
MSTO-211H
92.1
Mel202
Day 8
Day 15
Day 22
-Log10 (FDR)
b
H2052 (Mesothelioma)
Day 08
Day 15
Day 22
$y = -0.163 + 0.423\,x$ $R^2 = 0.11$
$y = -0.245 + 0.83\,x$ $R^2 = 0.65$
$y = -0.383 + 0.899\,x$ $R^2 = 0.73$
Primary screen Log2FC (vs Day 0)
Secondary screen Log2FC (vs Day 0)
92.1 (Uveal Melanoma)
$y = -0.238 + 0.969\,x$ $R^2 = 0.54$
$y = -0.351 + 1.09\,x$ $R^2 = 0.78$
$y = -0.52 + 1.1\,x$ $R^2 = 0.76$
c
TMM normalized counts correlation
Mel202_Day08_2
Mel202_Day08_1
92.1_Day08_2
92.1_Day08_1
NCI−H2052_Day08_2
NCI−H2052_Day08_1
Plasmid_d0
Mel202_Day15_2
Mel202_Day15_1
Mel202_Day22_2
Mel202_Day22_1
92.1_Day15_2
92.1_Day15_1
92.1_Day22_2
92.1_Day22_1
MSTO−211H_Day22_2
MSTO−211H_Day22_1
MSTO−211H_Day15_2
MSTO−211H_Day15_1
NCI−H2052_Day22_2
NCI−H2052_Day22_1
NCI−H2052_Day15_2
NCI−H2052_Day15_1
MSTO−211H_Day08_2
MSTO−211H_Day08_1
Cell line
NCI−H2052
MSTO−211H
92.1
Mel−202
Plasmid
Correlation
1.00
0.75
0.50
0.25
0.00
d
YAP1
TAZ
TEAD1
TEAD4
TEAD2
TEAD3
Log2FC
Day:
NCI-H2052
MSTO-211H
92.1
Mel202
Day 8
Day 15
Day 22

Figure S6

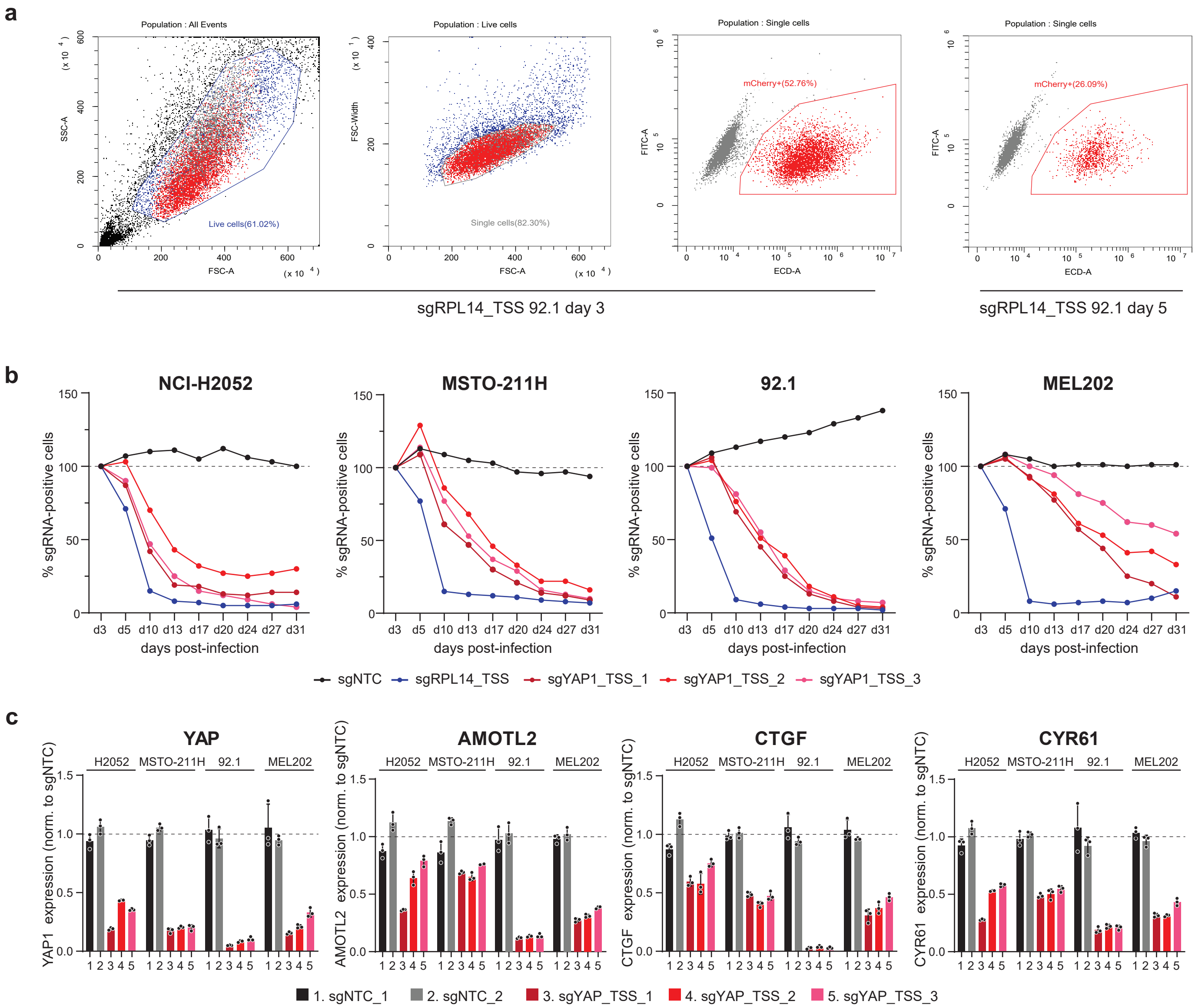
a
Population : All Events
Live cells(61.02%)
SSC-A
FSC-A
Population : Live cells
Single cells(82.30%)
FSC-Width
Population : Single cells
mCherry+(52.76%)
FITC-A
ECD-A
mCherry+(26.09%)
sgRPL14_TSS 92.1 day 3
sgRPL14_TSS 92.1 day 5
b
NCI-H2052
MSTO-211H
92.1
MEL202
% sgRNA-positive cells
days post-infection
d3 d5 d10 d13 d17 d20 d24 d27 d31
sgNTC
sgRPL14_TSS
sgYAP1_TSS_1
sgYAP1_TSS_2
sgYAP1_TSS_3
c
YAP
AMOTL2
CTGF
CYR61
H2052
MSTO-211H
92.1
MEL202
YAP1 expression (norm. to sgNTC)
AMOTL2 expression (norm. to sgNTC)
CTGF expression (norm. to sgNTC)
CYR61 expression (norm. to sgNTC)
1. sgNTC_1
2. sgNTC_2
3. sgYAP_TSS_1
4. sgYAP_TSS_2
5. sgYAP_TSS_3

Figure S6 (cont.)

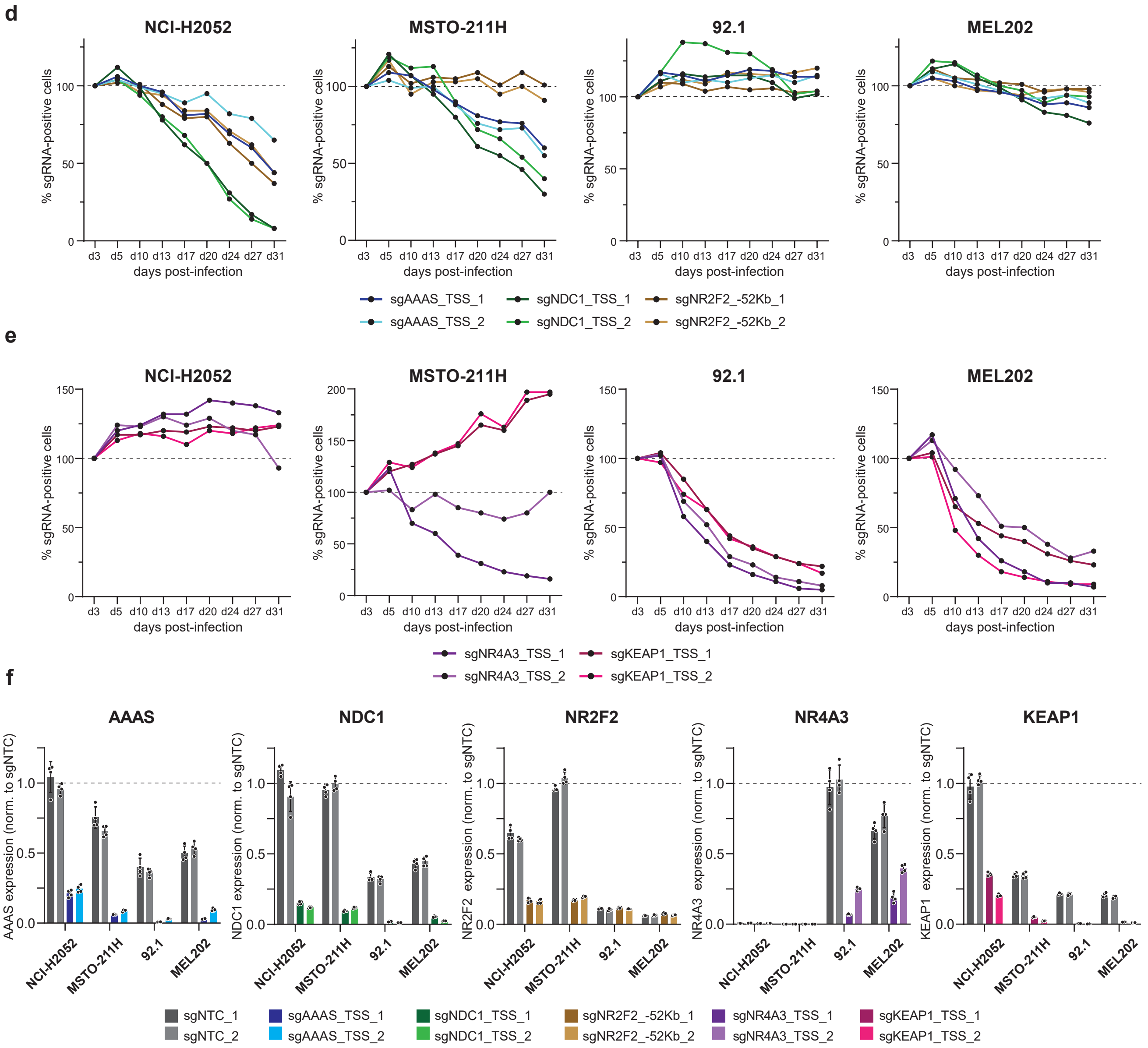

Figure S7

a

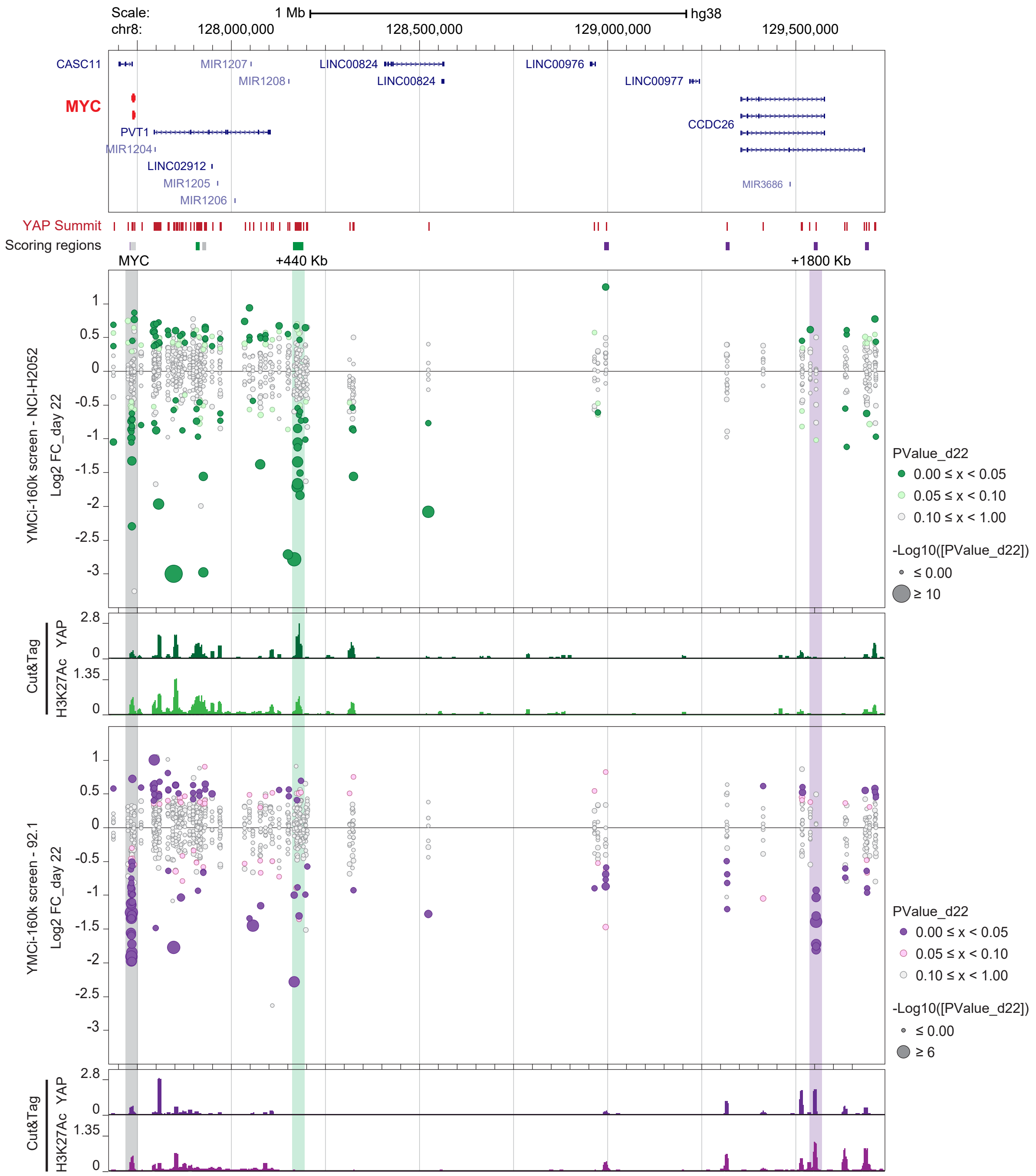

b

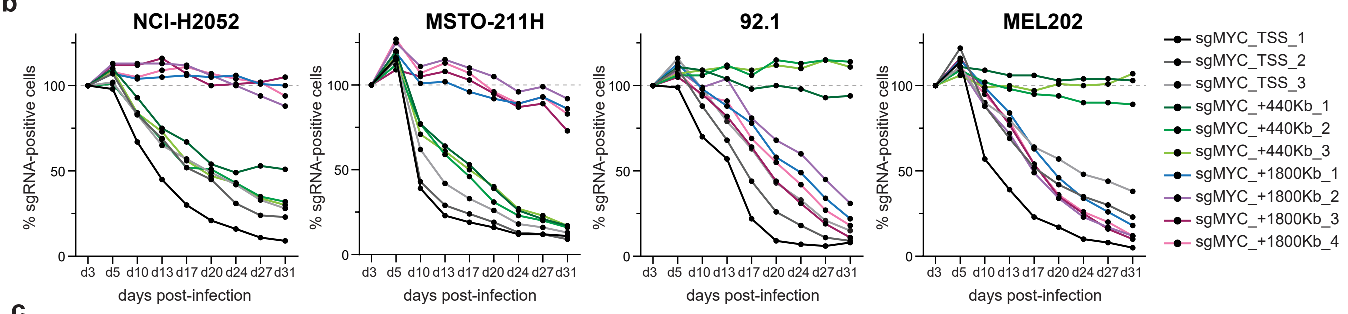

c

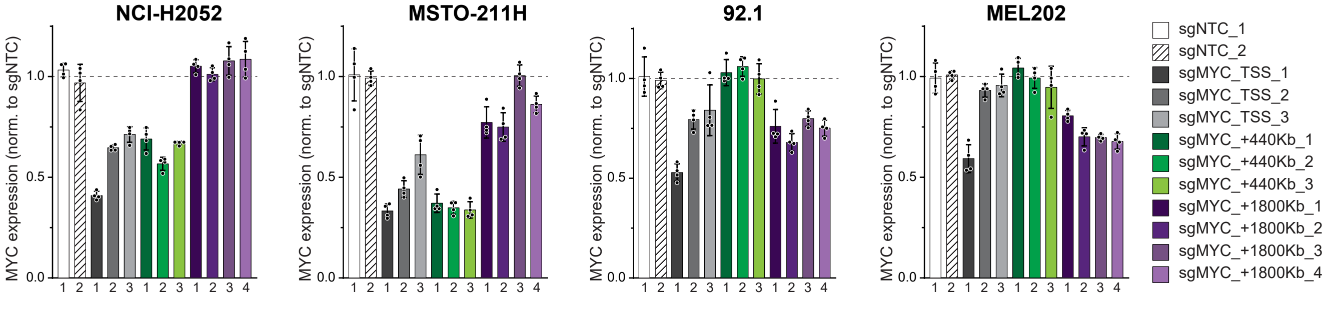

Figure S8

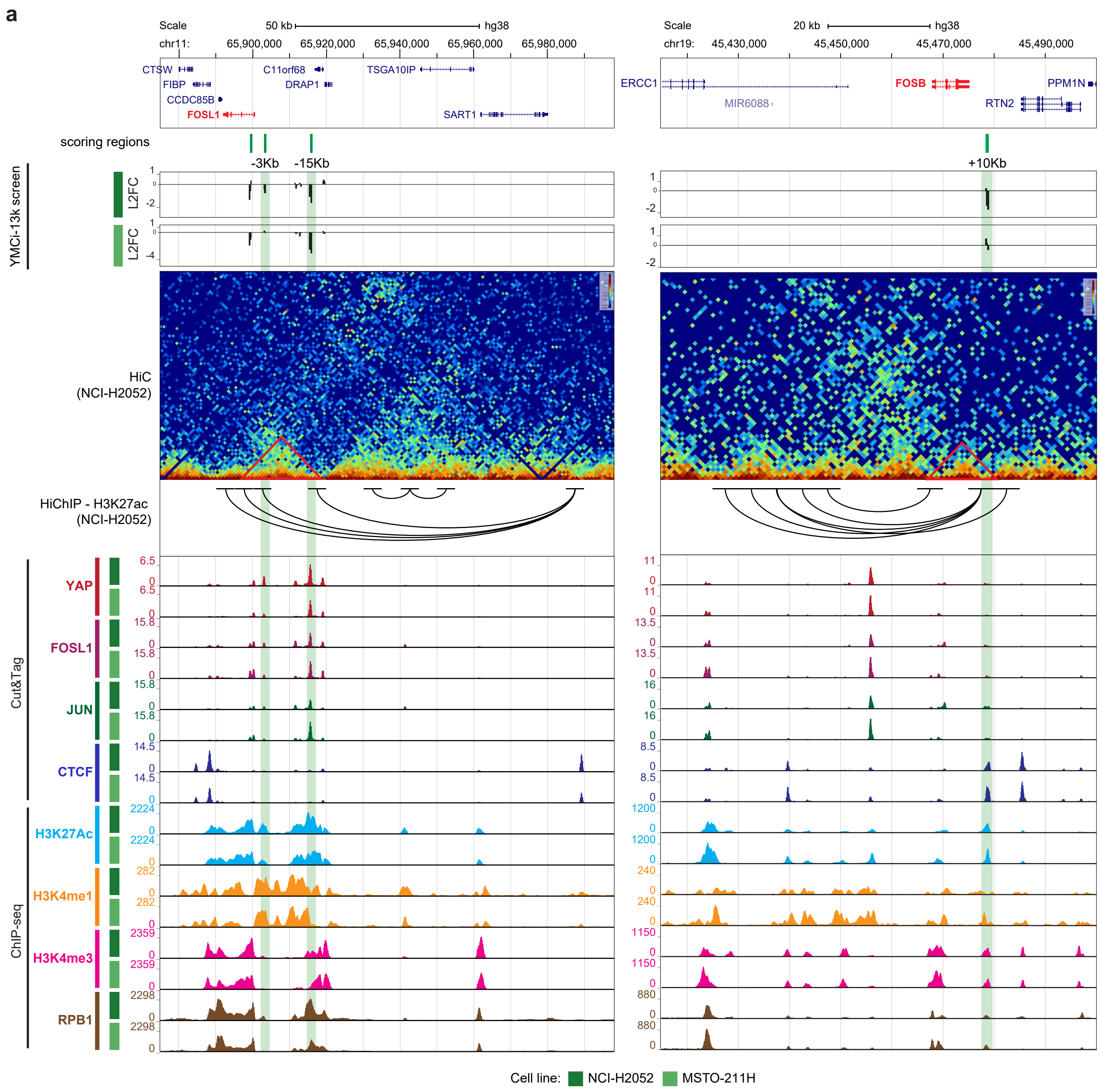

a
Scale
50 kb
hg38
chr11:
65,900,000
65,920,000
65,940,000
65,960,000
65,980,000
CTSW
FIBP
CCDC85B
FOSL1
C11orf68
DRAP1
TSGA10IP
SART1
Scale
20 kb
hg38
chr19:
45,430,000
45,450,000
45,470,000
45,490,000
ERCC1
MIR6088
FOSB
PPM1N
RTN2
scoring regions
-3Kb
-15Kb
+10Kb
YMCi-13k screen
L2FC
HiC
(NCI-H2052)
HiChIP - H3K27ac
(NCI-H2052)
Cut&Tag
YAP
FOSL1
JUN
CTCF
ChIP-seq
H3K27Ac
H3K4me1
H3K4me3
RPB1
Cell line: NCI-H2052 MSTO-211H

Figure S9

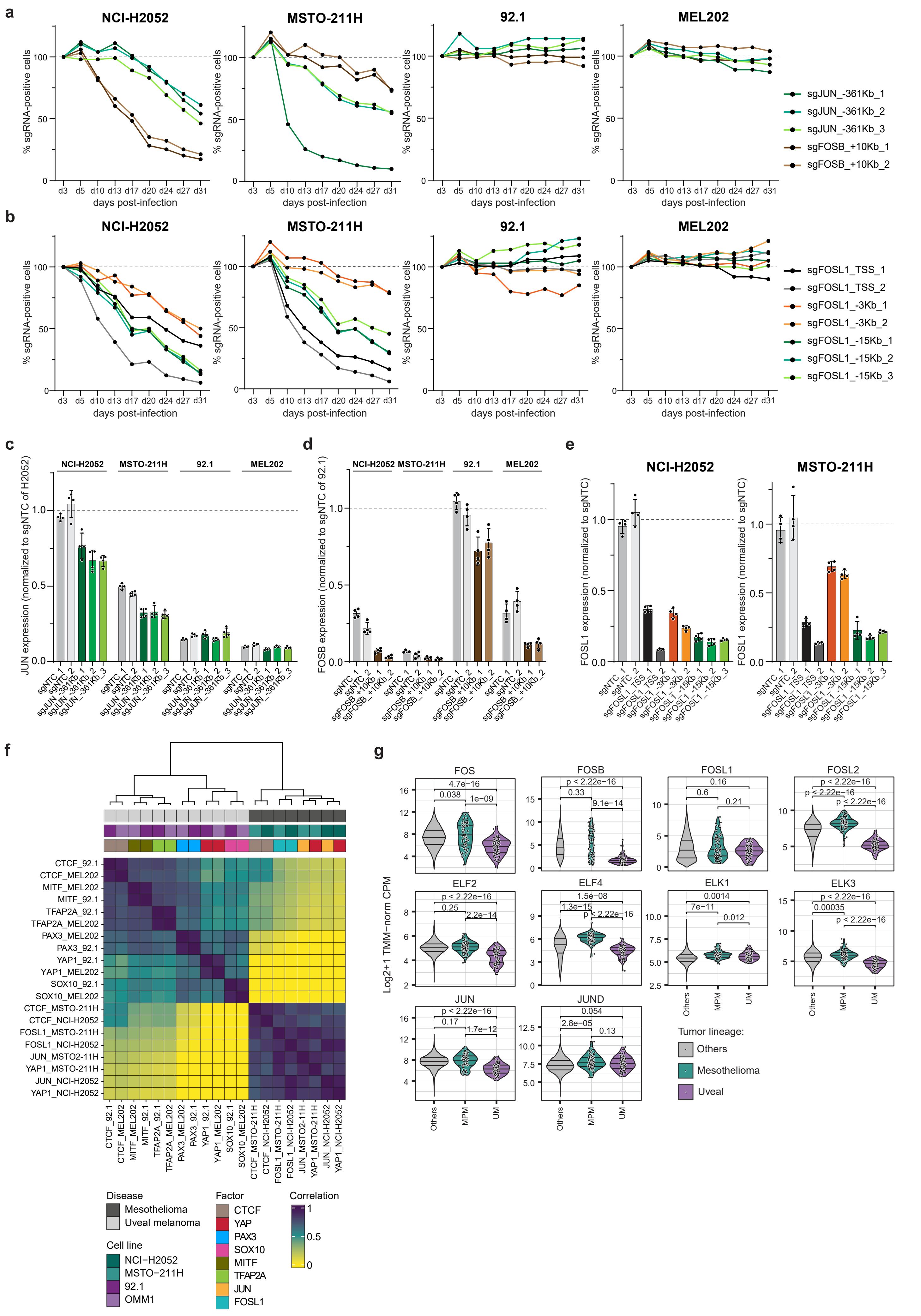
a
NCI-H2052
MSTO-211H
92.1
MEL202
% sgRNA-positive cells
days post-infection
sgJUN_-361Kb_1
sgJUN_-361Kb_2
sgJUN_-361Kb_3
sgFOSB_+10Kb_1
sgFOSB_+10Kb_2
b
sgFOSL1_TSS_1
sgFOSL1_TSS_2
sgFOSL1_-3Kb_1
sgFOSL1_-3Kb_2
sgFOSL1_-15Kb_1
sgFOSL1_-15Kb_2
sgFOSL1_-15Kb_3
c
JUN expression (normalized to sgNTC of H2052)
d
FOSB expression (normalized to sgNTC of 92.1)
e
FOSL1 expression (normalized to sgNTC)
f
Disease
Mesothelioma
Uveal melanoma
Cell line
NCI−H2052
MSTO−211H
92.1
OMM1
Factor
CTCF
YAP
PAX3
SOX10
MITF
TFAP2A
JUN
FOSL1
Correlation
g
FOS
FOSB
FOSL1
FOSL2
ELF2
ELF4
ELK1
ELK3
JUN
JUND
Log2+1 TMM−norm CPM
Tumor lineage:
Others
Mesothelioma
Uveal

Figure S10

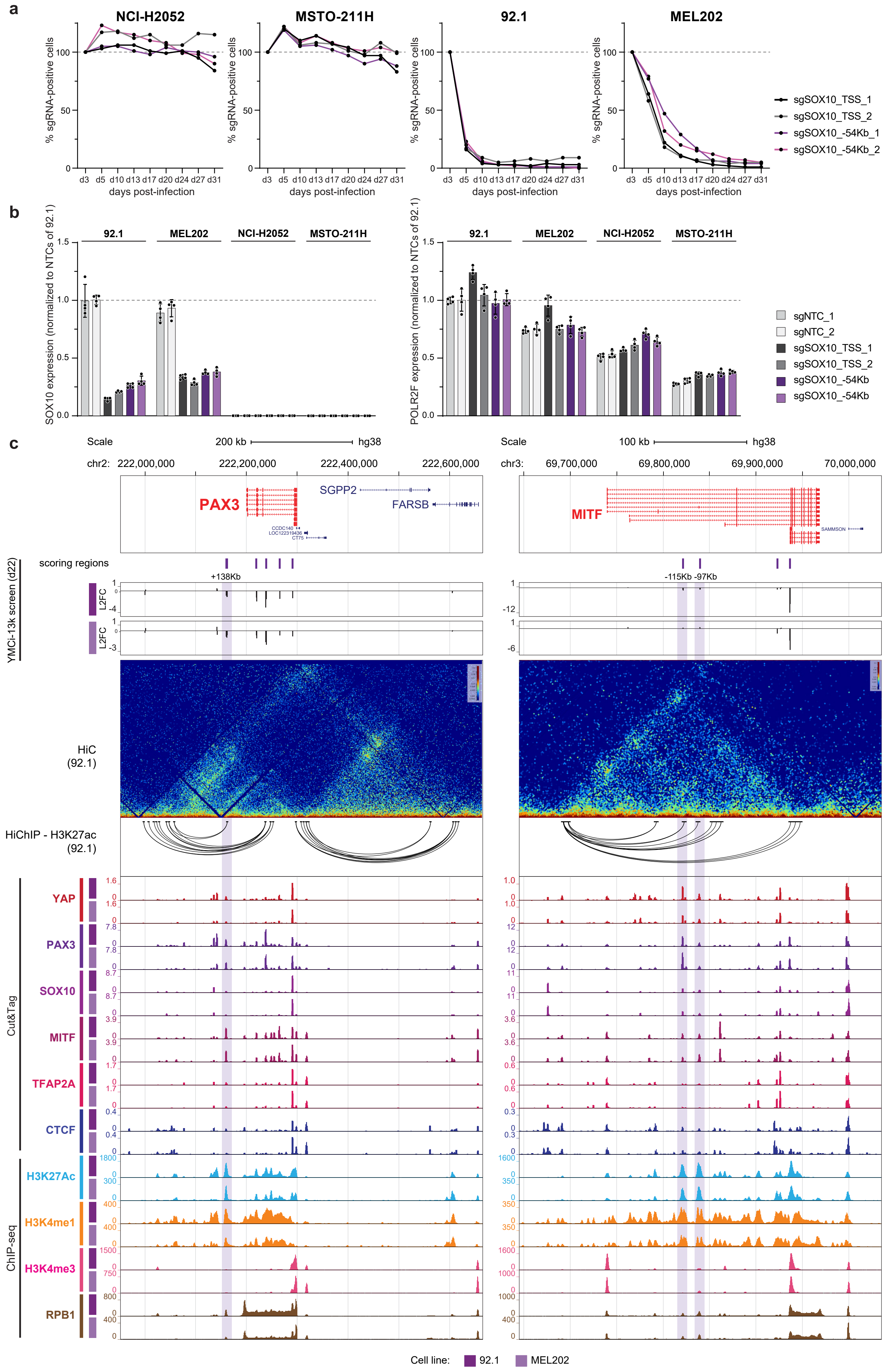

a
NCI-H2052
MSTO-211H
92.1
MEL202
% sgRNA-positive cells
days post-infection
d3 d5 d10 d13 d17 d20 d24 d27 d31
sgSOX10_TSS_1
sgSOX10_TSS_2
sgSOX10_-54Kb_1
sgSOX10_-54Kb_2
b
SOX10 expression (normalized to NTCs of 92.1)
POLR2F expression (normalized to NTCs of 92.1)
sgNTC_1
sgNTC_2
sgSOX10_TSS_1
sgSOX10_TSS_2
sgSOX10_-54Kb
sgSOX10_-54Kb
c
Scale
200 kb
hg38
chr2: 222,000,000
222,200,000
222,400,000
222,600,000
PAX3
SGPP2
FARSB
CCDC140
LOC122319436
CT75
100 kb
chr3: 69,700,000
69,800,000
69,900,000
70,000,000
MITF
SAMMSON
scoring regions
+138Kb
-115Kb -97Kb
YMCi-13k screen (d22)
L2FC
HiC (92.1)
HiChIP - H3K27ac (92.1)
Cut&Tag
YAP
PAX3
SOX10
MITF
TFAP2A
CTCF
ChIP-seq
H3K27Ac
H3K4me1
H3K4me3
RPB1
Cell line: 92.1 MEL202

Figure S10 (cont.)

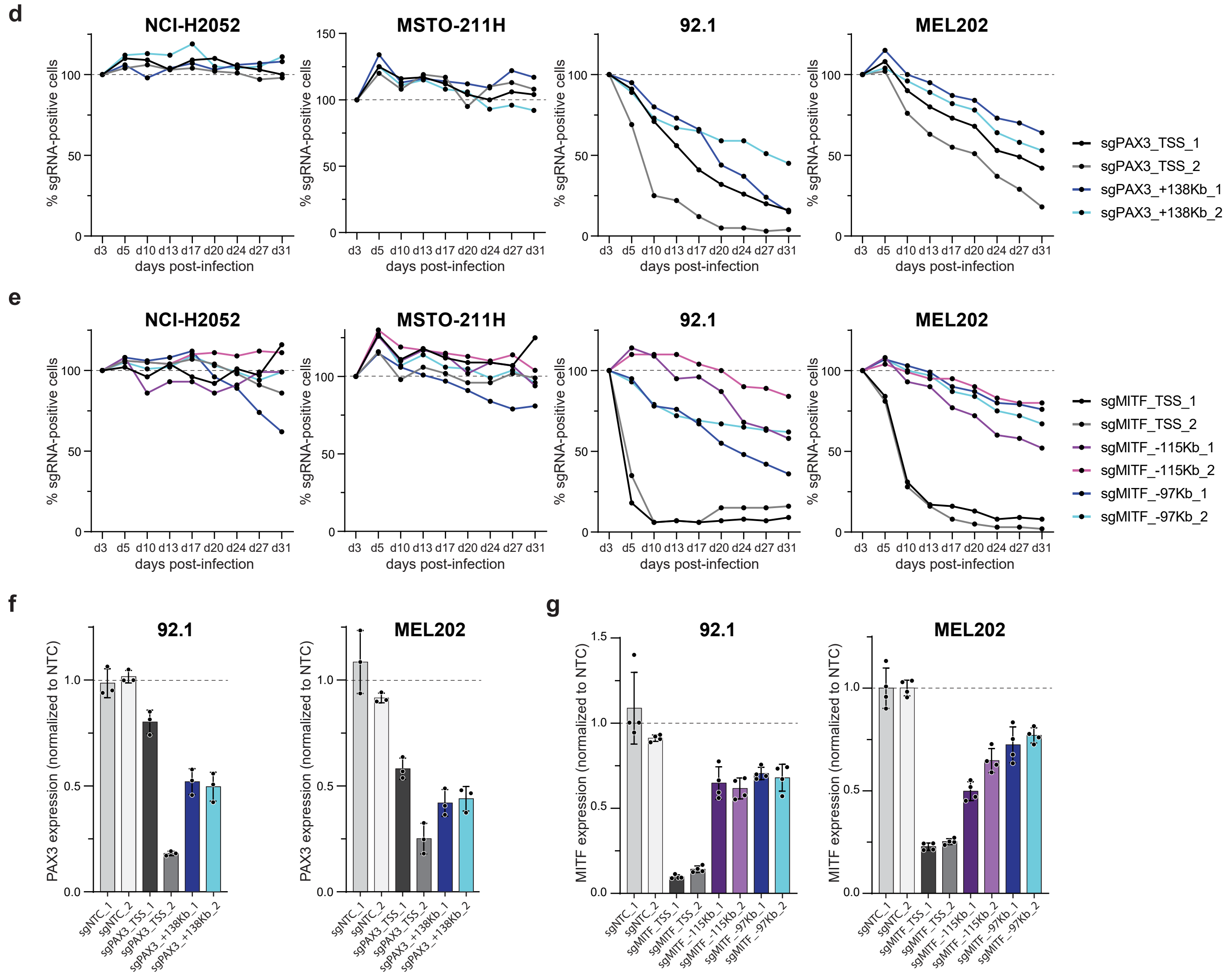

Figure S11

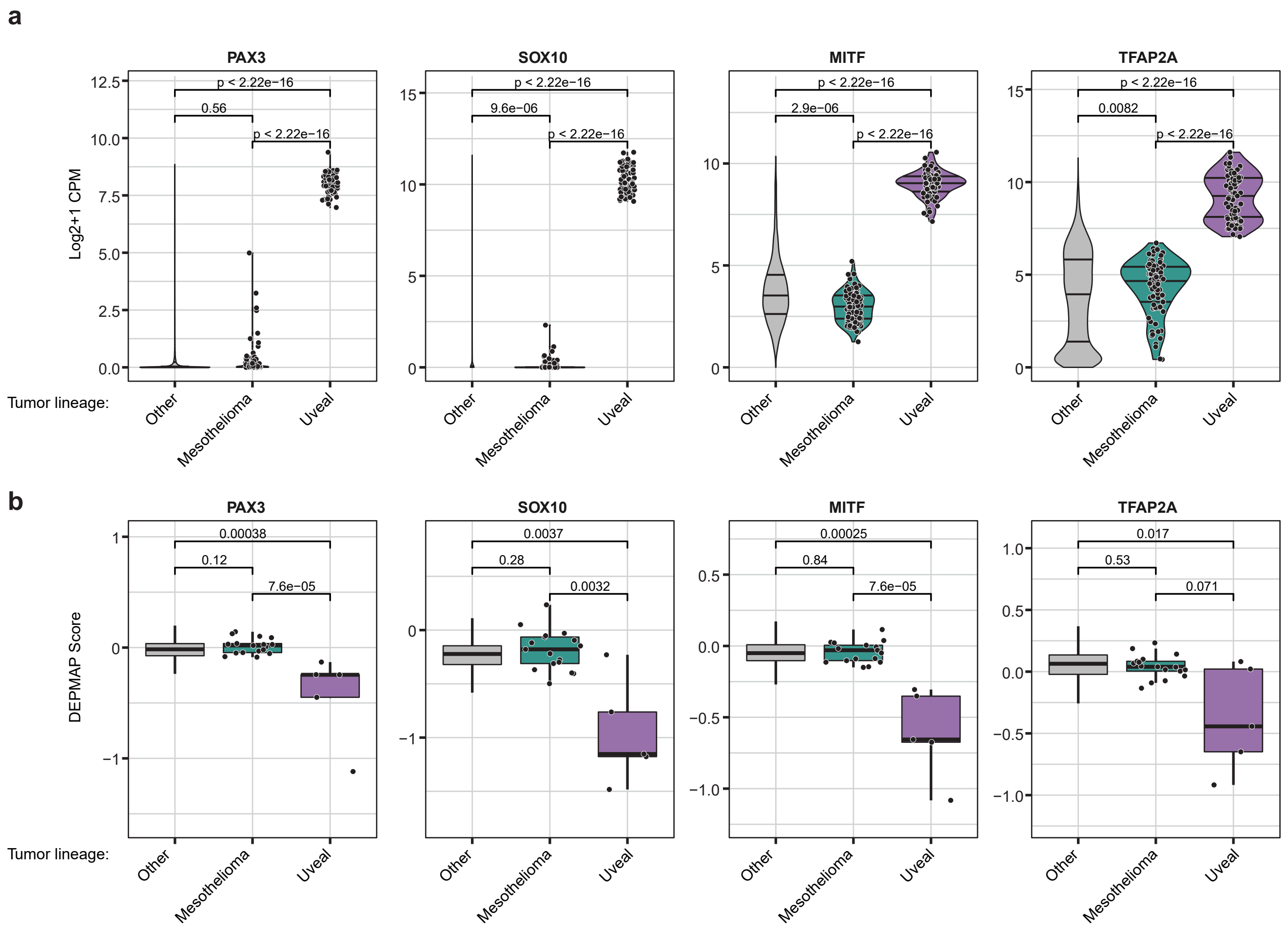

a
PAX3
SOX10
MITF
TFAP2A
Log2+1 CPM
p < 2.22e−16
0.56
9.6e−06
2.9e−06
0.0082
Tumor lineage:
Other
Mesothelioma
Uveal
b
DEPMAP Score
0.00038
0.12
7.6e−05
0.0037
0.28
0.0032
0.00025
0.84
0.017
0.53
0.071
