# Supplementary material for: Cancer lineage-specific regulation of YAP responsive elements revealed through large-scale functional epigenomic screens
Source: Nat Commun. 2023 Jul 3;14:3907. doi: 10.1038/s41467-023-39527-w (PMC10317959; doi:10.1038/s41467-023-39527-w)
Supplement: Supplementary file 4 — Description of Additional Supplementary Files [file 41467_2023_39527_MOESM4_ESM.pdf]

Description of Additional Supplementary Files

Supplementary Data 1: YMCi160K_LibraryFeatures

YMCi library containing approximately 160K sgRNAs targeting YAP-peak regions identified by ChIPseq in mesothelioma (NCI-H2052, MSTO-211H) and uveal melanoma (92.1 and OMM1) cell lines.

Supplementary Data 2: YMCi160K_Screen_RawCounts

Raw sequencing counts of YMCi-160K library (day 0) and screens in mesothelioma (NCI-H2052) and uveal melanoma (92.1) cell lines, for both replicates (r1 and r2) at days 8, 15 and 22.

Supplementary Data 3: YMCi160K_Screen_LogFC_representation

EdgeR analysis of YMCi-160K screen, showing Log Fold Changes, p-values and FDR values. This analysis was used to design the YMCi-13K library.

Supplementary Data 4: YMCi160K_Screen_SummitMAGECKanalysis

MAGECK analysis of YMCi-160K screen, combining behaviour of sgRNAs targeting the same YAPpeak summit regions. Values for negative- and positive-selection analyses are provided. This analysis was used for the transcription factor motif analysis shown in Figure 2D, using the scoring regions that compose the YMCi-13K library.

Supplementary Data 5: YMCi13K_LibraryFeatures

YMCi-13K sgRNA library containing 13000 sgRNAs targeting YAP-peak summit regions that scored in YMCi-160K screen in both cell lines (category 'Common') or in one cell line (Categories 'Meso' or 'Uveal').

Supplementary Data 6: YMCi13K_Screen_RawCounts

Raw sequencing counts of YMCi-13K library (day 0) and screens in mesothelioma (NCI-H2052 and MSTO-211H) and uveal melanoma (92.1 and MEL202) cell lines, for both replicates (r1 and r2) at 8, 15 and 22 days post-infection.

Supplementary Data 7: YMCi13K_Screen_analysis_FITNESS_SCORE_and_hit_calling

YMCi-13K screen analysis taking into account the behaviour overtime of groups of sgRNAs targeting YAP-peak summit regions.

Supplementary Data 8: YMCi13K_Screen_SummitMAGECKanalysis_HeatmapFIG3

MAGECK analysis of YMCi-13K screen, combining behaviour of sgRNAs targeting the same YAP-peak summit. Data is provided for all hits shown on heatmap of Figure 3C. Description of table content by column:

A, B: order of appearance in heatmap and scoring category to which the hit belongs (Common, UM-or MPM-specific);

C-F: genomic coordinates of YAP-peak summits and YMCi-library Union IDs;

G-I: annotation of closest TSS and distance to closest TSS (if the same TSS is annotated more than once it will be given a number – 1, 2, 3, …, n);

J, K: putative target assigned to screen-hits, based on inspection of Hi-C and Hi-ChIP loops, and distance to TSS of putative target gene.

L-CF: Values for negative-selection of MAGECK analysis.

Supplementary Data 9: Antibodies-Probes-sgRNAs-shRNAs

Detailed information on the reagents used in this study: antibodies used for ChIP-seq and Cut&Tag, qPCR probes, sgRNAs used in single validation experiments and shRNAs.
